# Synthesis and *In Vitro* Cytotoxic Activity of Chromenopyridones

**DOI:** 10.1155/2013/984329

**Published:** 2012-01-08

**Authors:** Balwinder Singh, Vishal Sharma, Gagandeep Singh, Rakesh Kumar, Saroj Arora, Mohan Paul Singh Ishar

**Affiliations:** ^1^Bio-Organic and Photochemistry Laboratory, Department of Pharmaceutical Sciences, Guru Nanak Dev University, Amritsar 143005, India; ^2^Botanical and Environmental Sciences, Guru Nanak Dev University, Amritsar 143005, India

## Abstract

Novel substituted chromenopyridones (**3a**–**j** and **6a**–**d**) were synthesized and evaluated *in vitro* for the cytotoxic activity against various human cancer cell lines such as prostate (PC-3), breast (MCF-7), CNS (IMR-32), cervix (Hela), and liver (Hep-G2). preliminary cytotoxic screening showed that all the compounds possess a good to moderate inhibitory activity against various cancer cell lines. Particularly, compound **6b** bearing allyl moiety displayed a significant cytotoxic potential in comparison to standard drugs.

## 1. Introduction 

Cancer is the most dreaded group of diseases in which abnormal cells divide aggressively without control invade and spread to other parts of the body through the blood and lymph systems [1]. Despite tremendous advancements both in early diagnosis and approaches to treatment, cancer still remains an unconquered problem [1]. The development of new chemotherapeutic agents for the treatment of cancer with fewer side effects is an important goal for medicinal chemists. Heterocyclic compounds display a high degree of structural diversity and constitute the largest, most varied family of organic compounds evaluated for anticancer potential in past decades [2]. A perusal of the literature revealed that chromone framework constitutes an integral part of several natural products and biologically active molecules. Chromone derivatives exhibit a remarkable spectrum of pharmacological activities including antitumor [3], anti-inflammatory [4], antibacterial [5], antifungal [6], antioxidant [7], anti-HIV [8], vasodilator [9], antiviral [10], and antiallergenic [11]. Naturally occurring chromone based molecule, morusin, isolated from a Chinese herb, has been reported to be a potent antitumor agent [12]. Recently, we have developed some chromone based compounds such as substituted 3-(5-phenyl-3*H*-[1,2,4]dithiazol-3-yl)chromen-4-one and substituted 4-oxo-4*H*-chromene-3-carbothioic acid *N*-phenylthioamide, which display a promising cytotoxic activity against a number of human cancer cell lines [13]. Derivatives of 6-chloro-/fluorochromone have also been reported as potential topoisomerase inhibitor anticancer agents [14]. 

2-Pyridone moiety is another substructural unit of several natural and synthetic molecules exhibiting diverse biological activities such as antitumor [15, 16], antimicrobial [17], anti-inflammatory [18], antiviral [19], and so forth. For instance, camptothecin, isolated from *Camptotheca acuminate* [20], is a naturally occurring DNA topoisomerase I poison possessing a pyridone nucleus. Due to a remarkable antiproliferative activity of camptothecin, various synthetic derivatives have been synthesized and among them, two water-soluble derivatives have gained FDA approval, that is, irinotecan to treat colorectal cancers and topotecan for ovarian and small-cell lung cancers [21]. Additionally, some camptothecinoids such as lurtotecan and exatecan are currently under clinical trials [22]. Keeping in view the high anticancer activity of both chromone and pyridones, it was decided to synthesize chromenopyridones and evaluate their cytotoxic potential against various human cancer cell lines.

## 2. Result and Discussion

**Scheme 1:**
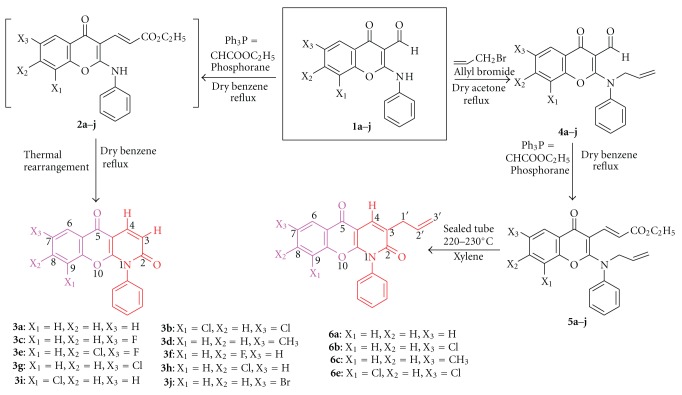
Synthesis of chromenopyridones **3a–j** and **6a–d**.

Variously substituted 2-anilino-3-formylchromones (**1a–j**) were reacted with ethoxycarbonylmethylene-triphenyl-phosphorane in dry benzene leading to the formation of intermediates (**2a–j**), which rearranged thermally on refluxing, yielding the corresponding substituted 1-phenyl-5-oxo-chromano[2,3-*b*]-dihydro-2[1*H*]-pyridones** (3a–j)**; the latter were purified by column chromatography over silica gel (60–120 mesh) using 8% ethyl acetate in hexane [23, 24]. All purified compounds were characterized by spectroscopic techniques (^1^H and ^13^C NMR, IR, mass) and elemental analysis (Scheme 1 and Table 1).

^1^H NMR spectra of compound **3b** showed two olefinic doublets, one at *δ* 8.20 and another at *δ* 6.90 ppm attributed to C_4_-H and C_3_-H, respectively, with coupling constant value 9.0 Hz. ^13^C NMR spectrum of **3b** showed resonance at *δ* 175.3 and *δ* 164.6 ppm attributed to the (C=O) group of both chromone and pyridone moiety, respectively, which was further corroborated by IR spectrum which showed strong bands at 1693 and 1687 cm^−1^. IR spectrum also showed C–N stretching at 1419 cm^−1^ of pyridone ring. The structure **3b** is further confirmed by mass spectrum which revealed a molecular ion peak at *m/z* 358 (M^+^).

 Keeping in view the high antitumor potential of isoprenyl/allyl substituted chromone derivatives [12–25], allyl substituted compounds **6a–d** were also synthesized. For the synthesis of latter, substituted 2-anilino-3-formylchromones (**1a–d**) were reacted with allyl bromide in the presence of fused potassium carbonate to obtain *N*-allylated products (**4a–d**); the latter on refluxing with ethoxycarbonylmethylene-triphenyl-phosphorane in dry benzene afforded Wittig product that is 2-(*N*-allyl)-3-(*E*-*β*-ethoxycarbonyl-ethenyl)-chromones (**5a–d**). When xylene solutions of **5a–d** were sealed in pyrex glass tubes and heated at 220–230°C for 6 hours, these thermally rearranged to substituted 3-allyl-1-phenyl-5-oxo-chromano[2,3-*b*]dihydro-2[1*H*]-pyridones (**6a–d**, Scheme 1 and Table 1). Compounds **6a–d** were purified by column chromatography over silica gel (60 : 120 mesh) using hexane and ethyl acetate in the ratio of 9 : 1 and characterized by spectroscopic techniques (^1^H and ^13^C NMR, IR, mass) and elemental analysis [24].

The ^1^H NMR spectra of compound **6b** shows the peaks at 6.01–5.93 (m, 1H, C_2′_-H), 5.22 (d, 1H, *J* = 11.4 Hz, C_3′_-H), 5.12 (d, 1H, *J* = 8.4 Hz, C_3′_-H), and 3.31 (d, 2H, *J* = 6.6 Hz, C_1′_-Hs) alluding to the presence of allyl moiety. Assigned structure is also supported by the presence of a singlet at *δ* 8.09 attributed to C_4_-H. The presence of 2-pyridone moiety in **6b** was established by a band in the IR spectrum around 1643 cm^−1^ and ^13^C NMR chemical shift of C_2_ (*δ* 160.6); overall ^13^C NMR assignments and mass spectra are in consonance with the assigned structures [24].

The cytotoxic potential of all the synthesized compounds **3a–j **and **6a–d** was observed against various human cancer cell lines such as prostate (PC-3), breast (MCF-7), CNS (IMR-32), cervix (Hela) and liver (Hep-G2) according to the procedure of Skehan et al. [26]. The growth inhibition (%) was determined in triplicate at concentrations 10, 30, 50, and 100 *μ*M/mL by using paclitaxel, 5-fluorouracil and mitomycin-C as a positive control. IC_50_ value (*μ*M), which is the concentration required to inhibit cancer cell proliferation by 50% after the exposure of cells to test compounds have also been determined (Table 2). In the case of prostate cancer cell line (PC-3), compound **6b** displayed promising cytotoxicity with IC_50_ = 2.4, followed by compounds **6c** and **6d** which showed moderate inhibitory potential with IC_50_ = 13.4 and 14.1, respectively. The compound **6b **and** 6c** showed a significant inhibitory activity against breast cancer cell line (MCF-7) with IC_50_ = 10.7 and 11.0, whereas compounds **6d** and **3h **showed good cytotoxicity with IC_50_ = 14.6 and 15.5 against the same cell line. In case of CNS cancer cell line (IMR-32), compounds **6b** and **6d** showed maximum inhibition with IC_50_ =13.2 and 16.3. Furthermore, in case of cervix cancer cell line (Hela) and liver cancer cell line (Hep-G2), compound **6b** showed maximum inhibitory activity with IC_50_ = 7.0 and 16.3, respectively. Perusal of the literature revealed that anticancer activities of chromone based compounds have been identified with various molecular mechanisms including carcinogen inactivation, antiproliferation, cell cycle arrest, induction of apoptosis and differentiation, inhibition of angiogenesis, antioxidant, and reversal of multidrug resistance [27]. Flavopiridol, derived from rohitukine, which is an alkaloidal-flavonoid present in the Indian plant *Dysoxylum binectariferum*, induces cell-cycle arrest at both G1 and G2 phases and is a potent inhibitor of CDK1, 2, 4, and 6 in a competitive manner with respect to ATP [28]. Quercetin, the natural flavonoid is reported to arrest the cell cycle and proliferation in prostate cancer cells by modulation of CDK1/Cyclin B; cell cycle analysis showed that it blocks G2-M transition with a significant induction of apoptosis [29]. Psorospermin is a chromone-based molecule possessing an epoxide group and has been reported to display a significant cytotoxic activity with topoisomerase II inhibitory potential. It is proposed that psorospermin's xanthone group intercalates with DNA and the epoxide ring undergoes alkylation at the N_7_-guanine base of DNA in the presence of topoisomerase II [30–32].

Though systemic establishment of the structure activity relationship has not been taken up, however, based on the presently observed cytotoxic activity of chromenopyridones against various human cancer cell lines, it emerges that compound bearing allyl moiety such as **6b** showed better cytotoxic activity than simple chromenopyridones. Compounds bearing electron withdrawing groups such as chloro and bromo at C_6_, C_7_, and C_8_ were found to be more active against cancer cells than compounds bearing an electron donating group. 

## 3. Conclusion

The chromenopyridones **3a–j** and **6a–d** were synthesized and evaluated for their cytotoxic activity against various human cancer cell lines. All compounds were purified with column chromatography and characterized by spectroscopic (^1^H and ^13^C NMR, IR, mass) and elemental analysis. The *in vitro* cytotoxic evaluation revealed that compounds bearing electron withdrawing groups on chromone moiety were more active against cancer cells than compounds bearing electron donating groups and allylated chromenopyridones (**6a–d**) displayed a higher activity. These “lead” compounds can be used for further anticancer drug development and their mode of action studies.

## 4. Experimental

### 4.1. Chemistry

Starting materials and reagents were purchased from commercial suppliers and used after further purification (crystallisation/distillation). JEOL AL-300FT (300 MHz) NMR spectrometer was used to record the ^1^H NMR and ^13^C NMR (75 MHz). Chemical shift (*δ*) and coupling constant are reported in ppm and Hz, respectively. Tetramethylsilane is used as the internal standard. IR spectra of compounds were recorded with shimadzu FT-IR 8400S spectrophotometer by using KBr pellets and CHCl_3_ as solvent. Mass spectra were recorded on Shimadzu GCMS-QP-2000A (EI method) and Bruker Daltonics Esquire 3000 (ESI-MS method) spectrometers.

### 4.2. Synthesis of Substituted 1-Phenyl-5-oxo-chromano[2,3-b]-dihydro-2[*1H*]-pyridones (**3a–j**)

To solutions of substituted 2-anilino-3-formylchromone (**1a–j**, 0.53 g, 2.0 mmol) in dry benzene (100 mL), ethoxycarbonylmethylene-triphenyl-phosphorane (0.7 g, 2.0 mmol) was added and the reaction mixtures were refluxed with stirring till the completion of reaction (12 h, TLC). The solvent was removed under vacuum so as to reduce the volume to about 10 mL and the solutions were kept under refrigeration until cream colored crystal separated. The crystals were filtered out and recrystallized from benzene to obtain the titled compounds as off-white solids [24].

### 4.3. Synthesis of Substituted 3-Allyl-1-phenyl-5-oxo-chromano[2,3-b]dihydro-2[*1H*]-pyridones (**6a–d**)

2-(*N*-Allyl-anilino)-3-(*E*-*β*-ethoxycarbonyl-ethenyl)-chromones (**5a–d**, 1.00 mmol) were dissolved in xylene (10 mL) and sealed in the glass tubes, which were heated at 220–230°C (6 h). The tubes were chilled and cut and solvents were evaporated under reduced pressure to 1/4 of the original volume. The crystal which appeared after being kept for two hours under refrigeration was filtered and recrystallized from benzene to obtain the substituted 3-allyl-1-phenyl-5-oxo-chromano [2,3-*b*]dihydro-2[1*H*]-pyridones (**6a–d**) as brown crystalline solids [24].

Spectral data of synthesized compounds **3a–j** and **6a–d**.

#### 4.3.1.  1-Phenyl-5-oxo-chromano[2,3-b]-dihydro-2[*1H*]-pyridone (**3a**)

Off-white solid, mp: 284–285°C; IR(CHCl_3_) *ν*_max_: 1678, 1663, 1647, 1630, 1600, 1560, 1517, 1481, 1458, 1404, 1353, 1281, 1224 cm^−1^; ^1^H NMR (CDCl_3_, 300 MHz): *δ* 8.20–8.14 (overlapping doublets, 2H, *J *= 9 Hz, C_4_ & C_6_-Hs), 7.60–7.20 (m, 7H, arom-Hs), 7.1 (d, 1H, *J *= 9 Hz, C_9_-H), 6.50 (d, 1H, *J *= 9.9 Hz, C_3_-H); ^13^C NMR (CDCl_3_, 75 MHz): *δ* 173.3 (C_5_), 161.6 (C_2_), 156.1 (C_10a_), 153.6 (C_9a_), 136.1 (CH), 133.9 (CH), 132.5 (N-Ph), 129.6 (CH), 129.2 (CH), 128.4 (CH), 126.7 (CH), 125.9 (CH), 122.2 (C_5a_), 117.5 (C_9_), 117.0 (CH), 102.3 (C_4a_); Mass *m/z*: 290 (M^+^+1), 289 (M^+^); Analysis: Calcd. for C_18_H_11_NO_3_: C 74.73; H 3.83; N 4.8%; Found: C 74.65; H 3.76; N 4.73%.

#### 4.3.2.  7,9-Dichloro-1-phenyl-5-oxo-chromano[2,3-b]-dihydro-2[*1H*]-pyridone (**3b**)

Off-white solid, mp: 286–287°C; IR(CHCl_3_) *ν*_max_: 1693, 1687, 1654, 1622, 1596, 1575, 1562, 1540, 1458, 1419, 1394, 1319, 1213 cm^−1^; ^1^H NMR (CDCl_3_, 300 MHz): *δ* 8.20 (d, 1H, *J *= 10.7 Hz, C_4_-H), 8.10 (d, 1H, *J *= 2.4 Hz, C_8_-H), 7.62–7.21 (m, 5H, arom-Hs), 6.61 (d, 1H, *J *= 10.0 Hz, C_3_-H); ^13^C NMR (CDCl_3_, 75 MHz): *δ* 175.3 (C_5_), 164.6 (C_2_), 157.2 (C_10a_), 155.3 (C_9_), 154.2 (C_7_), 153.6 (C_9a_), 138.5 (CH), 137.9 (CH), 136.5 (N-Ph), 132.6 (CH), 132.1 (CH), 131.4 (CH), 128.7 (CH), 126.9 (CH), 124.2 (C_5a_), 105.3 (C_4a_); Mass *m/z*: 358 (M^+^); Analysis: Calcd. for C_18_H_11_Cl_2_NO_3_: C 60.36; H 2.53; N 3.91%; Found: C 60.45; H 2.47; N 3.84%.

#### 4.3.3.  7-Flouro-1-phenyl-5-oxo-chromano[2,3-b]-dihydro-2[*1H*]-pyridone (**3c**)

Off-white solid,mp: 281–282°C; IR (CHCl_3_) *ν*_max_: 1685, 1657, 1642, 1627, 1608, 1554, 1522, 1475, 1456, 1415, 1345, 1274, 1230 cm^−1^; ^1^H NMR (CDCl_3_, 300 MHz): *δ* 7.92 (s, 1H, C_6_-H), 7.71 (d, 1H, *J* = 9.2 Hz, C_4_-H), 7.62-6.90 (m, 7H, arom-Hs), 6.64 (d, 1H, *J* = 9.6 Hz, C_3_-H); ^13^C NMR (CDCl_3_, 75 MHz): *δ* 172.1 (C_5_), 161.4 (C_2_), 155.9 (C_10a_), 151.8 (C_9a_), 134.0 (q), 133.8 (CH), 133.5 (N-Ph), 133.9 (CH), 131.6 (CH), 129.5 (CH), 128.3 (CH), 125.9 (CH), 123.2 (C_5a_), 119.1 (C_9_), 117.3 (CH), 102.0 (C_4a_); Mass *m/z*: 347 (M^+^+ K); Analysis: Calcd. for C_18_H_10_FNO_3_: C 70.36; H 3.28; N 4.56%; Found: C 70.42; H 3.26; N 4.63%.

#### 4.3.4.  7-Methyl-1-phenyl-5-oxo-chromano[2,3-b]-dihydro-2[*1H*]-pyridone (**3d**)

Off-white solid, mp: 287–288°C; IR (CHCl_3_)  *ν*_max_: 1679, 1658, 1637, 1622, 1589, 1562, 1540, 1458, 1436, 1394, 1340, 1320, 1294 cm^−1^; ^1^H NMR (CDCl_3_, 300 MHz): *δ* 8.0 (s, 1H, C_6_-H), 8.24 (d, 1H, *J *= 9.3 Hz, C_4_-H), 7.64-6.91 (m, 7H, arom-Hs), 6.53 (d, 1H, *J* = 9.3 Hz, C_3_-H), 2.41 (s, CH_3_-Hs); ^13^C NMR (CDCl_3_, 75 MHz): *δ* 170.3 (C_5_), 160.6 (C_2_), 155.1 (C_10a_), 152.6 (C_9a_), 136.8 (CH), 136.2 (C_7_), 135.6 (CH), 130.5 (N-Ph), 129.0 (CH), 129.2 (CH), 126.8 (CH), 126.71 (CH), 120.2 (C_5a_), 117.9 (C_9_), 117.09 (CH), 117.5 (C_4a_), 21.5 (CH_3_); Mass *m/z*: 304 (M^+^+ 1); Analysis: Calcd. for C_19_H_13_NO_3_: C 75.24; H 4.32; N 4.62%; Found: C 75.30; H 4.35; N 4.58%.

#### 4.3.5.  8-Chloro-7-flouro-1-phenyl-5-oxo-chromano[2,3-b]-dihydro-2[*1H*]-pyridone (**3e**)

Cream coloured solid, mp: 286–287°C; IR (CHCl_3_) *ν*_max_: 1697, 1676, 1649, 1580, 1540, 1510, 1496, 1446, 1396, 1342, 1260, 1215, 1116 cm^−1^; ^1^H NMR (CDCl_3_, 300 MHz): *δ* 8.28 (d, 1H, *J* = 8.7 Hz, C_4_-H), 7.65–7.10 (m, 7H, arom-Hs), 6.67 (d, 1H, *J* = 9.6 Hz, C_3_-H); ^13^C NMR (CDCl_3_, 75 MHz): *δ* 174.2 (C_5_), 163.6 (C_2_), 156.2 (C_10a_), 154.1 (C_9_), 153.1 (C_7_), 152.6 (C_9a_), 137.5 (CH), 137.1 (CH), 136.2 (N-Ph), 131.6 (CH), 131.1 (CH), 130.4 (CH), 128.3 (CH), 126.2 (CH), 123.1 (C_5a_), 103.2 (C_4a_); Mass *m/z*: 341.5 (M^+^+ Na); Analysis: Calcd. for C_18_H_9_ClFNO_3_: C 63.27; H 2.65; N 4.10%; Found: C 63.21; H 2.61; N 4.14%.

#### 4.3.6.  8-Flouro-1-phenyl-5-oxo-chromano[2,3-b]-dihydro-2[*1H*]-pyridone (**3f**)

Off-white solid, mp: 283–284°C; IR(CHCl_3_) *ν*_max_: 1685, 1654, 1625, 1537, 1488, 1438, 1400, 1369, 1321, 1260, 1232, 1182, 1114 cm^−1^; ^1^H NMR (CDCl_3_, 300 MHz): *δ* 8.21 (overlapping doublets, 2H, *J* = 9.6 Hz, C_4_ & C_6_-Hs),7.72–7.15 (m, 7H, arom-Hs), 6.65 (d, 1H, *J *= 9.3 Hz, C_3_-H); ^13^C NMR (CDCl_3_, 75 MHz): *δ* 171.0 (C_5_), 160.3 (C_2_), 155.2 (C_10a_), 153.7 (C_9a_), 135.3 (q-arom.), 132.9 (CH), 132.1 (N-Ph), 131.0 (CH), 130.7 (CH), 129.6 (CH), 127.2 (CH), 126.0 (CH), 121.2 (C_5a_), 118.1 (C_9_), 117.3 (CH), 101.4 (C_4a_); Mass *m/z*: 347 (M^+^+ K); Analysis: Calcd. for C_18_H_10_FNO_3_: C 70.36; H 3.28; N 4.56%; Found: C 70.31; H 3.19; N 4.47%.

#### 4.3.7.  7-Chloro-1-phenyl-5-oxo-chromano[2,3-b]-dihydro-2[*1H*]-pyridone (**3g**)

Off-white solid, mp: 285–286°C; IR (CHCl_3_) *ν*_max_: 1691, 1649, 1622, 1581, 1556, 1540, 1506, 1487, 1452, 1434, 1393, 1318, 1191 cm^−1^; ^1^H NMR (CDCl_3_, 300 MHz): *δ* 8.23(d, 1H, *J *= 8.4 Hz, C_4_-H), 7.63-7.06 (m, 8H, arom-Hs), 6.66 (d, 1H, *J *= 9.3 Hz, C_3_-H). ^13^C NMR (CDCl_3_, 75 MHz): *δ* 172.0 (C_5_), 161.3 (C_2_), 156.0 (C_10a_), 151.7 (C_9a_), 135.7 (q), 133.9 (CH), 133.4 (N-Ph), 132.0 (CH), 131.7 (CH), 129.6 (CH), 128.2 (CH), 126.0 (CH), 123.2 (C_5a_), 119.1 (C_9_), 117.3 (CH), 101.9 (C_4a_); Mass *m/z*: 349 (M^+^+Na+2); Analysis: Calcd. for C_18_H_10_ClNO_3_: C 66.78; H 3.11; N 4.33%; Found: C 66.71; H 3.15; N 4.37%.

#### 4.3.8.  8-Chloro-1-phenyl-5-oxo-chromano[2,3-b]-dihydro-2[*1H*]-pyridone (**3h**)

Off-white solid,mp: 286–287°C; IR (CHCl_3_) *ν*_max_: 1689, 1654, 1639, 1626, 1607, 1508, 1520, 1485, 1464, 1409, 1353, 1279, 1229 cm^−1^; ^1^H NMR (CDCl_3_, 300 MHz): *δ* 8.24 (overlapping doublets, 2H, *J* = 9.4 Hz, C_4_ & C_6_-Hs), 7.81–6.95 (m, 7H, arom-Hs), 5.82 (d, 1H, *J *= 9.5 Hz, C_3_-H); ^13^C NMR (CDCl_3_, 75 MHz): *δ* 172.0 (C_5_), 161.1 (C_2_), 156.8 (C_10a_), 150.9 (C_9a_), 135.2 (q), 133.5 (CH), 133.1 (N-Ph), 132.1 (CH), 131.8 (CH), 129.2 (CH), 128.1 (CH), 126.9 (CH), 123.1 (C_5a_), 118.1 (C_9_), 117.1 (CH), 101.4 (C_4a_); Mass *m/z*: 363 (M^+^+ K); Analysis: Calcd. for C_18_H_10_ClNO_3_: C 66.78; H 3.11; N 4.33%; Found: C 66.63; H 3.12; N 4.35%.

#### 4.3.9.  9-Chloro-1-phenyl-5-oxo-chromano[2,3-b]-dihydro-2[*1H*]-pyridone (**3i**)

Off-white solid, mp: 284–285°C; IR (CHCl_3_) *ν*_max_: 1693, 1649, 1627, 1610, 1579, 1540, 1495, 1469, 1446, 1396, 1346, 1290, 1265 cm^−1^; ^1^H NMR (CDCl_3_, 300 MHz): *δ* 8.21 (overlapping doublets, 2H, *J* = 9.6 Hz, C_4_ & C_6_-Hs), 7.83–7.08 (m, 7H, arom-Hs), 6.54 (d, 1H, *J *= 9.3 Hz, C_3_-H); ^13^C NMR (CDCl_3_, 75 MHz): *δ* 171.6 (C_5_), 162.0 (C_2_), 157.9 (C_10a_), 150.5 (C_9a_), 135.1 (q), 133.2 (CH), 133.0 (N-Ph), 131.0 (CH), 130.1 (CH), 129.1 (CH), 128.8 (CH), 126.2 (C_9_), 122.1 (C_5a_), 117.1 (CH), 101.1 (C_4a_); Mass *m/z*: 322 (M^+^+1); Analysis: Calcd. for C_18_H_10_ClNO_3_: C 66.78; H 3.11; N 4.33%; Found: C 66.59; H 3.05; N 4.29%.

#### 4.3.10.  7-Bromo-1-phenyl-5-oxo-chromano[2,3-b]-dihydro-2[*1H*]-pyridone (**3j**)

Off-white solid, mp: 288–289°C; IR (CHCl_3_) *ν*_max_: 1685, 1649, 1620, 1596, 1573, 1535, 1492, 1452, 1396, 1311, 1255, 1218, 1186 cm^−1^; ^1^H NMR (CDCl_3_, 300 MHz): *δ* 8.32 (s, 1H, C_6_-H), 8.10 (d, 1H, *J* = 9.9 Hz, C_4_-H), 7.52–7.05 (m, 7H, arom-Hs), 6.53 (d, 1H, *J* = 9.9 Hz, C_3_-H); ^13^C NMR (CDCl_3_, 75 MHz): *δ* 172.1 (C_5_), 161.3 (C_2_), 156.0 (C_10a_), 153.3 (C_9a_), 135.7 (CH), 133.9 (q), 133.4 (N-Ph), 132.0 (CH), 131.7 (CH), 129.6 (CH), 128.3 (CH), 126.0 (CH), 123.1 (C_5a_), 119.1 (C_9_), 117.3 (CH), 102.0 (C_4a_); Mass *m/z*: 368 (M^+^); Analysis: Calcd. for C_18_H_10_BrNO_3_: C 58.72; H 2.74; N 3.80%; Found: C 58.68; H 2.64; N 3.72%.

#### 4.3.11.  3-Allyl-1-phenyl-5-oxo-chromano[2,3-b]dihydro-2[*1H*]-pyridone (**6a**)

Brown crystalline solid, mp: 182–183°C; IR (CHCl_3_) *ν*_max_: 1674, 1649, 1614, 1583, 1552, 1481, 1468, 1450, 1427, 1410, 1390, 1344, 1290, 1267, 1190 cm^−1^; ^1^H NMR (CDCl_3_, 300 MHz): *δ* 8.24 (dd, 1H, *J *= 8.1 & 1.7 Hz, C_6_-H), 8.02 (s, 1H, C_4_-H), 7.54–7.20 (m, 7H, arom-Hs), 7.15 (d, 1H, *J* = 8.4 Hz, C_9_-H), 6.05–5.92 (m, 1H, C_2′_-H), 5.24 (d, 1H, *J* = 11.7 Hz, C_3′_ -H), 5.11 (d, 1H, *J* = 8.7 Hz, C_3′_-H), 3.33 (dd, 2H, *J* = 5.7 & 1.2 Hz, C_1′_-H); ^13^C NMR (CDCl_3_, 75 MHz): *δ* 173.6 (C_5_), 161.6 (C_2_), 154.7 (C_10a_), 153.4 (C_9a_), 134.3 (CH), 134.0 (N-Ph), 133.7 (CH), 132.0 (CH), 129.5 (CH), 129.4 (C_3_), 128.3 (CH), 128.2 (CH), 126.4 (CH), 125.6 (CH), 122.1 (C_5a_), 117.7 (C_9_), 117.4 (C_3_), 101.9 (C_4a_), 34.4 (C_1_); Mass *m/z*: 329 (M^+^); Analysis: Calcd. for C_21_H_15_NO_3_: C 76.58; H 4.59; N 4.25%; Found: C 76.51; H 4.53; N 4.21%.

#### 4.3.12.  3-Allyl-7-chloro-1-phenyl-5-oxo-chromano[2,3-b]dihydro-2v[*1H*]-pyridone(**6b**)

Brown crystalline solid, mp: 184–185°C; IR (CHCl_3_) *ν*_max_: 1676, 1643, 1617, 1580, 1542, 1496, 1450, 1430, 1396, 1350, 1380, 1225, 1272, 1219, 1180 cm^−1^; ^1^H NMR (CDCl_3_, 300 MHz): *δ* 8.12 (d, 1H, *J* = 2.1 Hz, C_6_-H), 8.02 (s, 1H, C_4_-H), 7.55–6.94 (m, 7H, arom-Hs), 6.01–5.93 (m, 1H, C_2′_-H), 5.22 (d, 1H, *J* = 11.4 Hz, C_3′_-H), 5.12 (d, 1H, *J* = 8.4 Hz, C_3′_-H), 3.31 (d, 2H, *J* = 6.6 Hz, *C*_1′_-Hs); ^13^C NMR (CDCl_3_, 75 MHz): *δ* 173.2 (C_5_), 160.6 (C_2_), 155.6 (C_10a_), 154.2 (q-arom.), 153.1 (C_9a_), 134.9 (CH), 134.4 (N-Ph), 132.8 (CH), 128.9 (CH), 128.2 (C_3_), 127.6 (CH), 127.1 (CH), 126.1 (CH), 125.7 (CH), 120.1 (C_5a_), 117.9 (C_9_), 117.3 (C_3_), 101.2 (C_4a_), 34.2 (C_1_); Mass *m/z*: 363.5 (M^+^); Analysis: Calcd. for C_21_H_14_ClNO_3_: C 69.33; H 3.88; N 3.85%; Found: C 69.27; H 3.78; N 3.82%.

#### 4.3.13.  3-Allyl-7-methyl-1-phenyl-5-oxo-chromano[2,3-b]dihydro-2[*1H*]-pyridone (**6c**)

Brown crystalline solid, mp: 186–188°C; IR (CHCl_3_) *ν*_max_: 1681, 1652, 1622, 1578, 1547, 1505, 1439, 1427, 1388, 1356, 1377, 1228, 1267, 1227, 1173 cm^−1^; ^1^H NMR (CDCl_3_, 300 MHz): *δ* 8.01 (d, 1H, *J* = 2.3 Hz, C_6_-H), 7.72 (s, 1H, C_4_-H), 7.63–6.91 (m, 7H, arom-Hs), 6.03–5.92 (m, 1H, C_2′_-H), 5.23 (d, 1H, *J* = 10.8 Hz, C_3′_-H), 5.14 (d, 1H, * J* = 9.4 Hz, C_3′_-H), 3.3 (d, 2H, *J* = 6.4 Hz, C_1′_-Hs), 2.3 (s, 3H, CH_3_-Hs); ^13^C NMR (CDCl_3_, 75 MHz): *δ* 170.2 (C_5_), 161.6 (C_2_), 155.1 (C_10a_), 152.1 (C_9a_), 149.2 (q-arom.), 134.4 (N-Ph), 132.8 (CH), 129.4 (CH), 128.2 (CH), 127.9 (C_3_), 127.1 (CH), 126.8 (CH), 126.0 (CH), 124.7 (CH), 120.4 (C_5a_), 117.5 (C_9_), 117.1 (C_3_), 100.2 (C_4a_), 34.1 (C_1_), 22.5 (CH_3_); HR-MS (ESI-TOF): 363.3400 (M^+^+ Na); Analysis: Calcd. for C_22_H_17_NO_3_: C 76.95; H 4.99; N 4.08%; Found: C 76.87; H 4.92; N 4.02%.

#### 4.3.14.  3-Allyl-7,9-dichloro-1-phenyl-5-oxo-chromano[2,3-b]dihydro-2[*1H*]-pyridone (**6d**)

Brown crystalline solid, mp: 186–187°C; IR (CHCl_3_) *ν*_max_: 1684, 1649, 1627, 1582, 1552, 1511, 1443, 1434, 1395, 1363, 1373, 1222, 1274, 1221, 1176 cm^−1^; ^1^H NMR (CDCl_3_, 300 MHz): *δ* 8.1 (d, 1H, *J* = 2.4 Hz, C_6_-H), 8.0 (s, 1H, C_4_-H), 7.5–6.9 (m, 6H, arom-Hs), 6.0–5.9 (m, 1H, C_2′_-H), 5.2 (d, 1H, *J* = 9.7 Hz, C_3′_-H), 5.2 (d, 1H, *J* = 10.3 Hz, C_3′_-H), 3.3 (d, 2H, *J* = 6.6 Hz, C_1′_-Hs); ^13^C NMR (CDCl_3_, 75 MHz): *δ* 173.7 (C_5_), 164.5 (C_2_), 155.5 (q-arom.), 154.5 (C_10a_), 153.1 (C_9a_), 149.2 (q-arom.), 134.7 (N-Ph), 133.5 (CH), 130.8 (CH), 129.9 (CH), 128.1 (C_3_), 127.5 (CH), 126.1 (CH), 125.1 (CH), 122.7 (CH), 120.4 (C_5a_), 117.9 (C_9_), 117.4 (C_3_), 103.2 (C_4a_), 34.5 (C_1_); HR-MS (ESI-TOF): 420.030 (M^+^+Na−1); Analysis: Calcd. for C_21_H_13_Cl_2_NO_3_: C 63.34; H 3.29; N 3.52%; Found: C 63.28; H 3.22; N 3.48%.

### 4.4. Biological Activity

The sulforhodamine B (SRB) assay is a colorimetric assay used for cytotoxic screening to assess cell growth [26]. Cells are cultured in a 96-well tissue culture plates and the cell growth which depends upon the rate of multiplication is measured indirectly by the intensity of the color of the dye which is directly proportional to the number of cells present. The human cancer cell lines (procured from NCI, Frederick, USA) were grown in tissue culture flasks in complete growth medium (RPMI-1640 medium with 2 mM glutamine, pH 7.4, supplemented with 10% fetal calf serum, 100 *μ*g/mL streptomycin, and 100 units/ml penicillin) in a carbon dioxide incubator (37°C, 5% CO_2_, 90% RH). The cells at subconfluent stage were harvested from the flask by treatment with trypsin (0.05% in PBS (pH 7.4) containing 0.02% EDTA). Cells with viability of more than 98% as determined by trypan blue exclusion were used for the determination of cytotoxicity and the cell suspension of 1 × 10^5^ cells/mL was prepared in a complete growth medium. Stock solutions (2 × 10^−2^ M) of synthesized compounds **3a–j** and **6a–d** were prepared in DMSO. The stock solutions were serially diluted with a complete growth medium containing 50 *μ*g/mL of gentamycin to obtain working test solutions of required concentrations. *In vitro *cytotoxicity against four human cancer cell lines of different tissues was determined using 96-well tissue culture plates. The 100 *μ*L of cell suspension was added to each well of the 96-well tissue culture plate. The cells were allowed to grow in a carbon dioxide incubator (37°C, 5% CO_2_, 90% RH) for 24 hours. Test materials in complete growth medium (100 *μ*L) were added after 24 hours of incubation to the wells containing cell suspension. The plates were further incubated for 48 hours in a carbon dioxide incubator. The cell growth was stopped by gently layering trichloroacetic acid (50%, 50 *μ*L) on top of the medium in all the wells. The plates were incubated at 4°C for one hour to fix the cells attached to the bottom of the wells. The liquid of all the wells was gently pipetted out and discarded. The plates were washed five times with distilled water to remove trichloroacetic acid, growth medium low molecular weight metabolites, serum proteins, and so forth, and were air-dried. The plates were stained with sulforhodamine B dye (0.4% in 1% acetic acid, 100 *μ*L) for 30 minutes. The plates were washed five times with 1% acetic acid and then air-dried. The adsorbed dye was dissolved in Tris-HCl buffer (100 *μ*L, 0.01 M, pH 10.4) and plates were gently stirred for 10 minutes on a mechanical stirrer. The optical density (OD) was recorded on ELISA reader at 540 nm. The cell growth was determined by subtracting the mean OD value of respective blank from the mean OD value of experimental set. Percent growth in presence of the test material was calculated considering the growth in the absence of any test material as 100% and in turn percent growth inhibition in presence of the test material was calculated.

## Figures and Tables

**Table 1:** Reaction time and yield (%) of various compounds **3a**–**j** and **6a**–**d**.

Compound number	X_1_	X_2_	X_3_	Reaction time (h)	Yield (%) of products
**3a**	H	H	H	12	90
**3b**	Cl	H	Cl	12	92
**3c**	H	H	F	12	92
**3d**	H	H	CH_3_	12	95
**3e**	H	Cl	F	12	89
**3f**	H	F	H	12	90
**3g**	H	H	Cl	12	93
**3h**	H	Cl	H	12	95
**3i**	Cl	H	H	12	94
**3j**	H	H	Br	12	95
**6a**	H	H	H	6	96
**6b**	H	H	Cl	6	94
**6c**	H	H	CH_3_	6	95
**6d**	Cl	H	Cl	6	95

**Table 2:** IC_50_ (*μ*M) of synthesized compounds against various human cancer cell lines.

Compound number	IC_50_ ± SE∗ in *μ*M				
	PC-3 (Prostate)	MCF-7 (Breast)	IMR-32 (CNS)	Hela (Cervix)	Hep-G2 (Liver)
**3a**	82.4 ± 3.5	90.6 ± 2.5	80.4 ± 3.5	>100	>100
**3b**	79.2 ± 3.5	80.2 ± 3	>100	>100	90.6 ± 2.5
**3c**	88.9 ± 2.3	>100	90.4 ± 3.5	88.8 ± 1.3	>100
**3d**	78.2 ± 2.4	86.2 ± 3.5	77.4 ± 3.5	>100	83.5 ± 1.2
**3e**	81.9 ± 2.6	83.2 ± 1.5	93.2 ± 2.5	>100	>100
**3f**	28.7 ± 3.3	39.6 ± 3.5	23.9 ± 2.8	15.7 ± 3.5	>100
**3g**	21.5 ± 2.5	23.8 ± 3.2	19.2 ± 2.3	20.4 ± 1.5	44.1 ± 2.1
**3h**	41.9 ± 3.5	15.5 ± 2.3	>100	28.7 ± 1.5	85.6 ± 2.2
**3i**	80.3 ± 1.3	54.6 ± 3.8	33.7 ± 1.2	21.8 ± 3.5	54.2 ± 2.6
**3j**	54.2 ± 2.5	33.4 ± 1.2	19.2 ± 2.5	88.2 ± 3.2	38.9 ± 1.1
**6a**	19.5 ± 2.4	>100	33.5 ± 3.3	36.1 ± 2.1	44.9 ± 3.2
**6b**	2.4 ± 3.4	10.7 ± 2.5	13.2 ± 2.3	7.0 ± 3.5	16.3 ± 3.5
**6c**	13.4 ± 1.1	11.0 ± 2.3	15.6 ± 2.1	41.9 ± 3.5	23.6 ± 2.5
**6d**	14.1 ± 2.2	14.6 ± 1.5	16.3 ± 1.4	>100	41.0 ± 2.3

Paclitaxel	**—**	**0.2 ± 0.03**	**—**	**—**	**—**
5-Fluorouracil	**—**	**—**	**1.3 ± 0.01**	**—**	**0.5 ± 0.05**
Mitomycin-C	**1.5 ± 0.02**	**—**	**—**	**2.0 ± 0.03**	**—**

∗Standard error (±).
